# Editorial: Multi-scale systems: ecological approaches to investigate the role of the microbiota in different niches

**DOI:** 10.3389/fmolb.2025.1665390

**Published:** 2025-08-08

**Authors:** Padhmanand Sudhakar, Kristel Van Steen, Amirul Islam Mallick, Kaline Arnauts

**Affiliations:** ^1^ Department of Biotechnology, Kumaraguru College of Technology, Coimbatore, Tamil Nadu, India; ^2^ BIO3 - Systems Genetics, GIGA Molecular & Computational Biology, Universite de Liege, Liège, Belgium; ^3^ Department of Biological Sciences, Indian Institute of Science Education and Research Kolkata, Mohanpur, West Bengal, India; ^4^ Department of Chronic Diseases and Metabolism (CHROMETA), Translational Research Center for Gastrointestinal Disorders (TARGID), KU Leuven, Leuven, Belgium

**Keywords:** microbiota, microbiome, omic data integration, systems biology, biomarker discovery, microbial functions

Last decade’s rapid development of new technologies (such as Next-Generation Sequencing technologies (Reuter et al., 2015; Lightbody et al., 2019), enabling high-throughput molecular profiling) have underscored the role of microbial communities and their coordinated interactions within complex ecosystems. Niches in which such microbial ecosystems exist range from the majority of anatomical sites of the human and animal body to the deepest depths of the oceans, previously thought to be devoid of life itself (Yu et al., 2019; Dinan et al., 2015). Through our Research Topic titled “Multi-Scale Systems: Ecological Approaches to Investigate the Role of the Microbiota in Different Niches” hosted by Frontiers in Molecular Biosciences, we have attempted to highlight the systemic nature of microbiota, their diversity and activity, in particular in both health and disease conditions.

Since microbes coexist and interact within different ecological niches present in various kingdoms of life (Yu et al., 2019; Dinan et al., 2015; Gupta et al., 2021), integration of available datasets representing such interactions (Lapatas et al., 2015; Gomez-Cabrero et al., 2014) seemed of paramount importance. These strategies not only help build more representative models of biological reality but also aid amongst others in discovering the molecular mechanisms (Sudhakar et al., 2022), biomarkers (Zeng et al., 2016), key molecules/hubs (Li X. et al., 2019; He et al., 2014) which could drive phenotypically essential aspects such as response to therapeutic regimens (Chiu et al., 2018; Iorio et al., 2015; Chen et al., 2016), exploring disease heterogeneity in clinical settings (Sudhakar et al., 2021), amenability to biological interventions to ameliorate environmental degradation (Li L. et al., 2019; Ayilara and Babalola, 2023), susceptibility to biotic/abiotic stress (Gupta et al., 2021; Braga et al., 2016Braga et al., 2016), and disease resistance (Vannier et al., 2019).

Over the past decade or two, various tools and approaches (Pic et al., 2021; Meng et al., 2016; Rohart et al., 2017; Ruffalo et al., 2015) emerged for integrating high-throughput molecular-omic datasets. As a part of our Research Topic, the study by Agamah et al., demonstrated how an integrative approach fusing different -omics signatures such as transcriptomics, metabolomics, proteomics, and lipidomics with disease phenotypes revealed a cross-panel network of molecules (a.k.a. the interactome) driving different phases of COVID-19 associated disease severity. In particular, the interactome representative of mild COVID-19 cases was characterized by hubs such as CCL4, IRF1, HGF, MMP12, and IL10. In contrast, severe COVID-19 cases were characterized by a completely different hub set, including STAT1, SOD2, and metabolites such as diacylglycerol, lysophosphatidylcholine, taurine, sphingomyelin, and triglycerides. In a similar study by Wang and Lv., submitted to our Research Topic, the authors discovered causal associations between gut microbial taxa and metabolites derived from plasma to the progression of asthma. Multi-layered high-throughput profiling-based generation of -omic datasets enabled these findings, while their integration can leverage disease state-specific hubs and mechanisms for the discovery of novel drugs and drug targets.

Yet another challenge in addressing the complexity of microbial systems is the variation between individual samples and interpreting the biological significance of that variation with regards to their effects on phenotypic manifestations. In this insightful article by Melograna et al., they explored how Individual Specific Networks (ISNs) can be constructed from faecal microbiome profiles of patients with Inflammatory Bowel Disease (IBD) undergoing various biological therapies. The reverse-engineered ISNs from a population of subjects were able to capture the microbiome-based features predictive of response, but also network structures representing microbial interactions, which were associated with response to the therapeutic regimens under consideration.

From a real-world perspective, long-term studies provide enhanced data richness by capturing latent effects, which are particularly prevalent in microbe-rich niches subject to complex exposomic and environmental factors. Hence, long-term studies enable the identification of microbial shifts, including the nature of these shifts in terms of diversity, the temporal validity of biomarkers, and environmental drivers that promote alterations in composition. The study by Li et al., in our topic collection, demonstrates the efficacy of long-term sampling strategies, especially for diseases such as COVID-19, which have a highly dynamic nature due to the interplay of various factors, including diet, immune system, medications, co-infections, and comorbidities. In particular, the authors demonstrate that mild COVID-19 infections, even after recovery, have lasting impacts on the gut microbiota, as evidenced by the enrichment of probiotic taxa, including *Blautia massiliensis* and *Kluyveromyces spp*. three months post-recovery.

Last but not least, mechanistic discoveries add depth to studies by integrating microbiome-derived datasets with individual or combined -omic datasets. The studies by Tan et al., and Nie et al., demonstrate the utility of using large datasets and integrating them with curated phenotypic data to uncover key macromolecules associated with the phenotype of interest, or that could potentially mechanistically drive the phenotype. For example, Nie et al., uncovered increased susceptibility to IBD by using mice harbouring somatic mutations in the gene encoding EpCAM, a protein found in the basolateral membrane of Intestinal Epithelial Cells (IECs). By simulating colitis development via administration of dextran sulfate sodium (DSS) in both wild-type mice and mice with EpCAM deficiencies, followed by host inflammatory markers analysis as well as profiling of gut microbial alterations, the authors were able to pinpoint a set of gene-based and microbial markers associated with the link between EpCAM mutation and colitis development.

In line with the potential of high-throughput profiling technologies to generate microbial datasets and integrative -omic techniques to fuse such datasets with other -omic data types, the articles in our Research Topic collection have highlighted several possibilities, albeit the discovery of biomarkers, understanding mechanisms of pathogenesis, host response to microbial infections or uncovering temporal patterns in response to environmental stimuli. We hope such discoveries ignite renewed interest in the scientific community, as well as law/policymakers and the public to investigate further the roles played by microbes in health as well as disease across different scales - from the planet to the people and everything in between.
